# Tmprss2 Is Essential for Influenza H1N1 Virus Pathogenesis in Mice

**DOI:** 10.1371/journal.ppat.1003774

**Published:** 2013-12-05

**Authors:** Bastian Hatesuer, Stephanie Bertram, Nora Mehnert, Mahmoud M. Bahgat, Peter S. Nelson, Stefan Pöhlman, Klaus Schughart

**Affiliations:** 1 Department of Infection Genetics, Helmholtz Centre for Infection Research, University of Veterinary Medicine Hannover, Braunschweig, Germany, and University of Tennessee Health Science Center, Memphis, Tennessee, United States of America; 2 Infection Biology Unit, German Primate Center, Göttingen, Germany; 3 Divisons of Human Biology and Clinical Research, Fred Hutchinson Cancer Research Center, Seattle, Washington, United States of America; Mount Sinai School of Medicine, United States of America

## Abstract

Annual influenza epidemics and occasional pandemics pose a severe threat to human health. Host cell factors required for viral spread but not for cellular survival are attractive targets for novel approaches to antiviral intervention. The cleavage activation of the influenza virus hemagglutinin (HA) by host cell proteases is essential for viral infectivity. However, it is unknown which proteases activate influenza viruses in mammals. Several candidates have been identified in cell culture studies, leading to the concept that influenza viruses can employ multiple enzymes to ensure their cleavage activation in the host. Here, we show that deletion of a single HA-activating protease gene, *Tmprss2*, in mice inhibits spread of mono-basic H1N1 influenza viruses, including the pandemic 2009 swine influenza virus. Lung pathology was strongly reduced and mutant mice were protected from weight loss, death and impairment of lung function. Also, after infection with mono-basic H3N2 influenza A virus body weight loss and survival was less severe in *Tmprss2* mutant compared to wild type mice. As expected, *Tmprss2-*deficient mice were not protected from viral spread and pathology after infection with multi-basic H7N7 influenza A virus. In conclusion, these results identify TMPRSS2 as a host cell factor essential for viral spread and pathogenesis of mono-basic H1N1 and H3N2 influenza A viruses.

## Author Summary

Seasonal influenza epidemics and pandemics represent a serious health threat to the human population. Resistance to presently available anti-viral drugs is frequently observed. Therefore, identification of new targets for anti-viral therapy is an urgent need. Host proteases are required for processing of the virus hemagglutinin and may thus represent a suitable target for intervention. Here, we report that the deletion of a single host protease gene, *Tmprss2*, in mice protects the host against viral spread in infected lungs. Only very mild pathogenesis was observed in *Tmprss2* mutant mice after infection with H1N1 virus and less severe pathogenesis was observed after infection with H3N2 virus. Thus, our results suggest that the host protease TMPRSS2 may be a prime target for antiviral intervention.

## Introduction

Annual influenza epidemics and unpredictable pandemics pose a severe threat to human health, exemplified by the estimated 30–50 million deaths caused by the 1918 pandemic. Current therapy targets viral proteins, neuraminidase and M2, but is hampered by development of resistance [1], due to the high mutation rate of the virus. Novel antiviral strategies are urgently required and invariable host cell factors essential for viral spread are attractive targets.

The cleavage of the influenza virus hemagglutinin (HA) by host cell proteases is essential for viral infectivity [2], [3]. The HA proteins of highly pathogenic avian influenza viruses harbor multiple basic amino acids at their cleavage site and are activated by furin [4]. In contrast, low pathogenic avian and human influenza viruses contain a mono-basic cleavage site in their HA proteins. Several studies showed that multiple secreted proteases can activate human influenza viruses for infection of cell lines (see [5], [6] for examples and [7] for a review). However, the analysis of cultured human respiratory epithelium demonstrated that influenza virus cleavage activation is a cell-associated process and no evidence for a role of secreted proteases was obtained [8]. Subsequently, the type II transmembrane serine protease (TTSP) family member TMPRSS2, a membrane associated protease, was shown to activate HA proteins of diverse human influenza viruses in cell culture [9], [10], [11], [12]. In addition, TMPRSS2 was found to be expressed in human respiratory epithelium positive for alpha 2,6-linked sialic acid [13]. However, the role of TMPRSS2 in influenza virus spread and pathogenesis in the infected host has not yet been studied.

Therefore, we investigated if TMPRSS2 contributes to influenza virus replication and pathogenesis in experimentally infected mice. We focused our analysis on viruses of the H1N1 (including the pandemic 2009 influenza virus) and H3N2 subtypes, since viruses of these subtypes are presently circulating in the human population. Our study shows that deletion of *Tmprss2* in knock-out mice strongly limits virus spread and lung pathology after H1N1 influenza A virus infection. The deletion of *Tmprss2* also reduces body weight loss and mortality after H3N2 infection but to a much lower degree than for H1N1 infected mice.

## Results

### Tmprss2 is essential for spread and pathogenesis of H1N1 influenza viruses in mice

**Figure 1:**
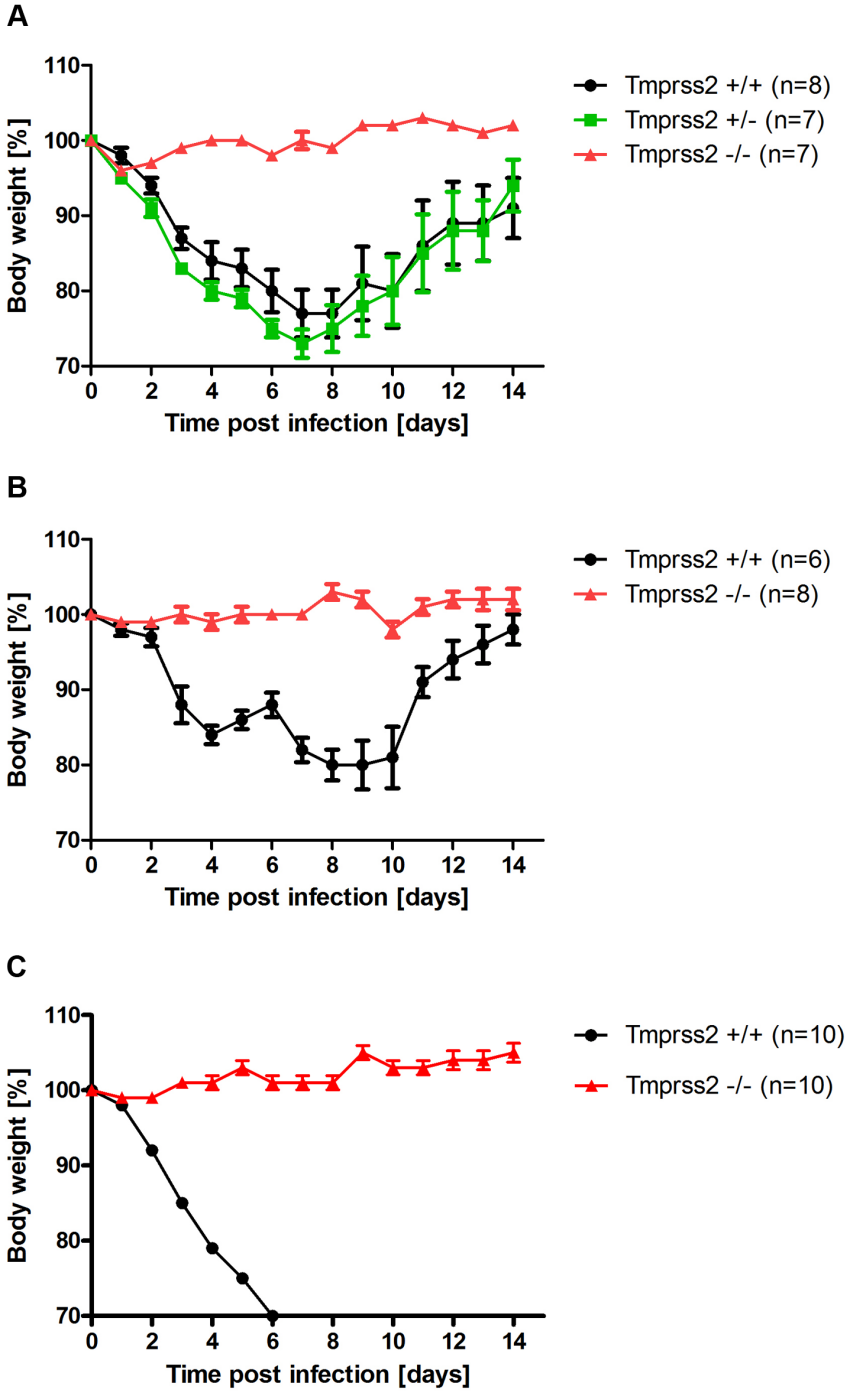
*Tmprss2* is essential for spread and pathogenesis of H1N1 influenza viruses. Eight to eleven weeks old female mice were infected with 2×10^5^ FFU mouse-adapted PR8M (H1N1; A), 2×10^5^ FFU HA4 (pH1N1, B), 2×10^3^ FFU mouse-adapted PR8F (H1N1; C). Body weight loss was monitored until day 14 p.i. Mice with a weight loss of more than 30% of the starting bodyweight were euthanized and recorded as dead. Weight loss data represent mean values +/− SEM. Note that only data of surviving mice are presented (*e.g.* about 50% of infected mice died after infection with PR8M, see Fig. S1). Body weight loss was significantly different between wild type and homozygous mutant mice at day 6 p.i. (p<0.0001 for PR8M infected mice, and p<0.0001 for HA4 infected mice, using the non-parametric Mann Whitney U test) and between heterozygous and homozygous mutant mice (PR8M infected mice, p<0.0001 using the Mann Whitney U test).

**Figure 2:**
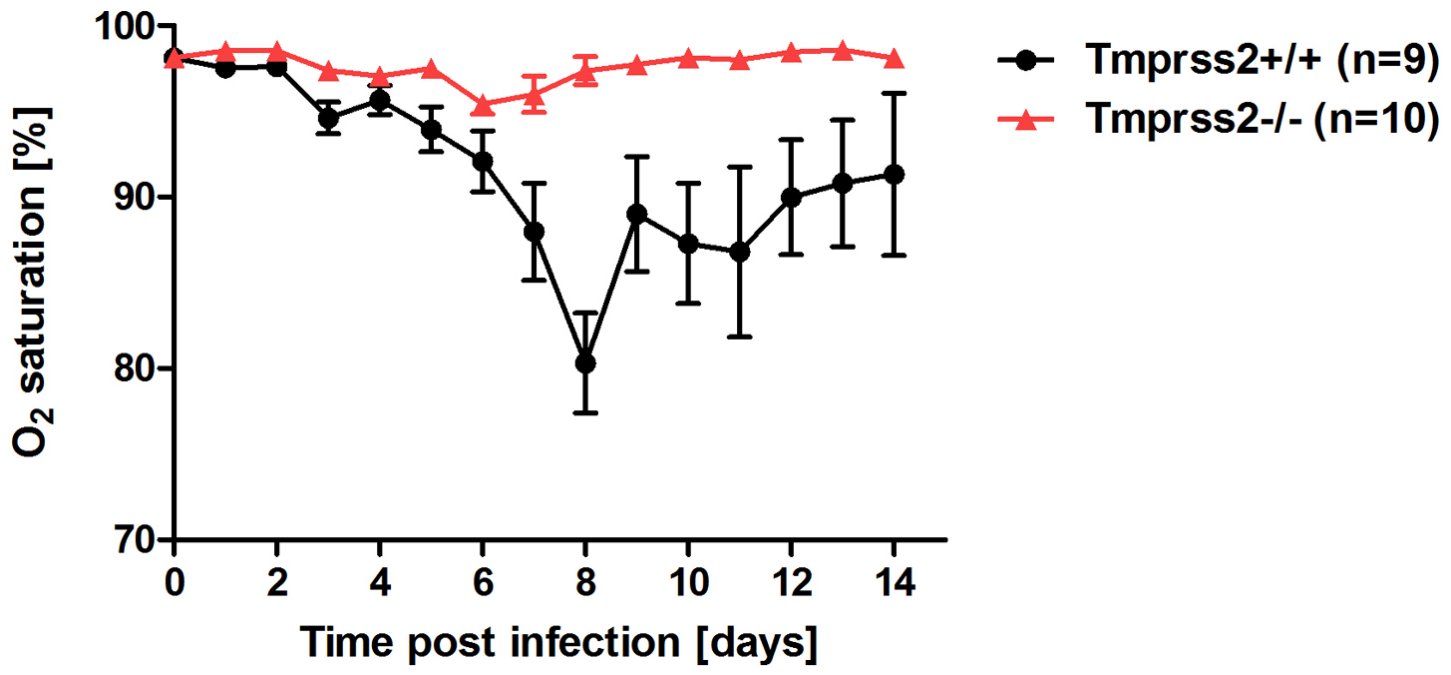
*Tmprss2^−/−^* mutant mice do not exhibit drop in blood oxygen saturation. *Tmprss2^−/−^* and wild type mice were infected with 2×10^5^ FFU PR8M virus and oxygen saturation in the peripheral blood was measured until day 14 p.i. *Tmprss2^−/−^* mice showed only a very mild drop in oxygen saturation whereas wild type mice exhibited a significant decrease that peaked at day 8 p.i.

To assess the role of TMPRSS2 during influenza virus infection *in vivo*, we used mice carrying a deletion of the *Tmprss2* gene [14]. Non-infected *Tmprss2* knock-out mice did not show a phenotype in the absence of infection, as described previously [14] and RT-PCR analysis of kidney tissue confirmed the absence of full length *Tmprss2* transcripts. Upon intranasal infection of mice with mouse-adapted PR8M (A/PuertoRico/8/34 H1N1 Münster variant, [15]), wild type mice lost weight significantly after infection and 50% of infected mice died, whereas *Tmprss2* knock-out mice did not exhibit body weight loss and showed no signs of disease (Figs. 1A; Fig. S1). The same results were obtained after infection with a human isolate of the pandemic HA4 (A/Hamburg/4/2009 H1N1) virus (Figure 1B). Also, after infection with a lethal dose of the more virulent PR8F virus isolate (A/PuertoRico/8/34 H1N1 Freiburg variant) all wild-type mice died within seven days post infection whereas no knock-out mice showed symptoms of disease (Figure 1C). Similar results were observed for blood oxygen saturation levels which provide a measurement for lung function. Whereas wild type mice exhibited a significant drop in peripheral blood oxygen saturation that peaked at day 8 post infection (p.i.) with PR8M virus, *Tmprss2^−/−^* mutant mice showed only a very mild change (Figure 2).

**Figure 3:**
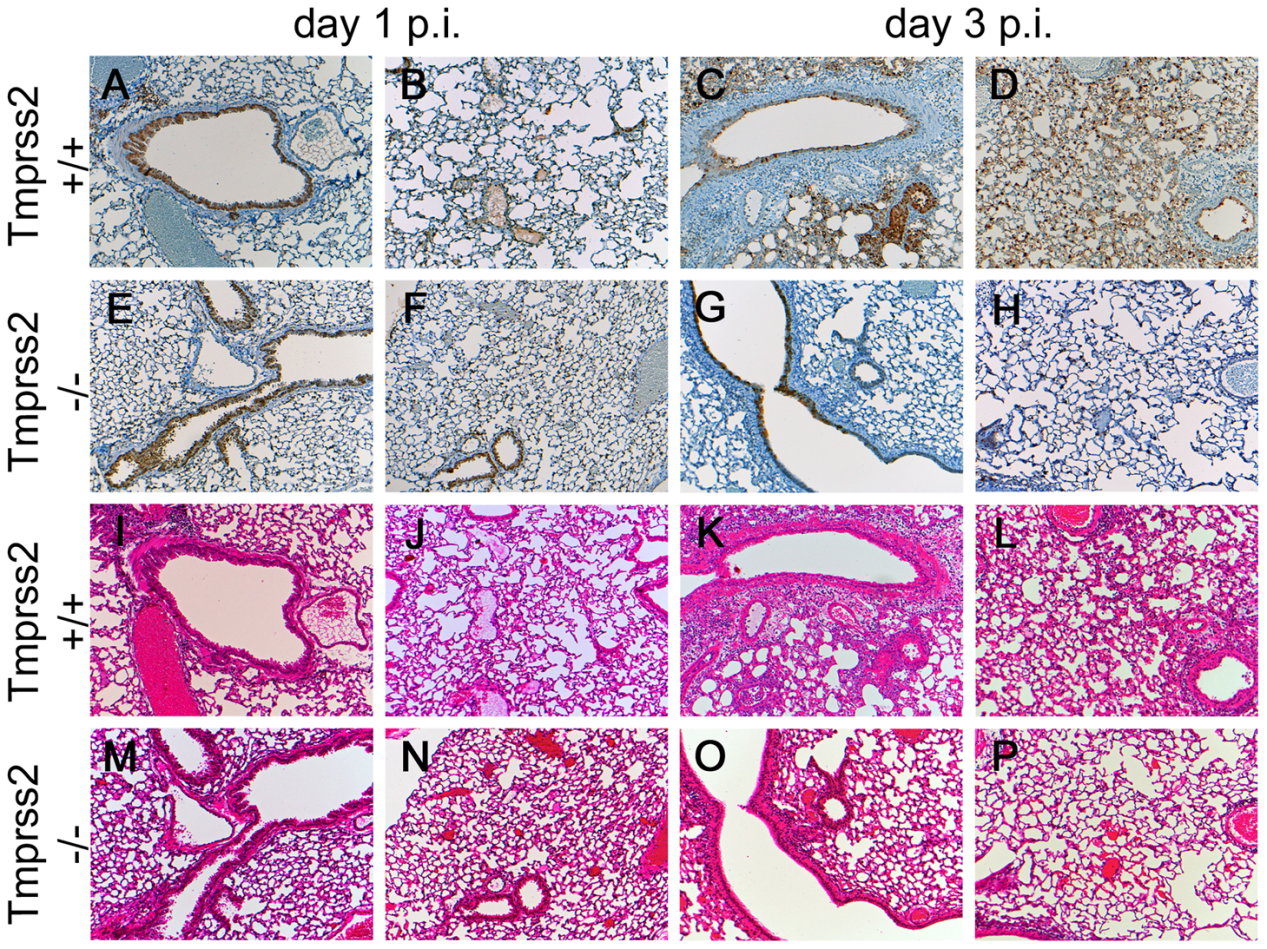
Mild lung pathology and reduced viral spread is observed in *Tmprss2^−/−^* mutant mice. Eight to twelve weeks old mice were infected intra-nasally with 2×10^5^ FFU of PR8M virus. Serial lung sections were stained at day 1 and day 3 p.i. with anti-influenza antibody and haematoxylin (A–H) or with haematoxylin/eosin (I–P). The overall lung tissues were more densely consolidated with larger numbers of infiltrating immune cells in wild type (I–L) compared to *Tmprss2^−/−^* mice (M–P). In addition, the airways of *Tmprss2^+/+^* mice were surrounded by higher numbers neutrophils and macrophages (I–L) whereas airways of *Tmprss2^−/−^* mice showed lower numbers of immune cell infiltrations (M–P). Virus-infected cells at day 1 p.i. were observed mainly in bronchiolar regions in the lungs of both mice (A, B, E, F). Both wild type and mutant mice showed influenza-positive cells at day 3 p.i. (C, G). However, the overall number of infected cells was lower in *Tmprss2^−/−^* compared to wild type mice. Furthermore, infected cells were mostly limited to bronchiolar regions in *Tmprss2^−/−^* whereas in wild type mice the virus also spread significantly into the alveolar regions (D, H).

Histological analyses of infected lungs revealed a similar onset of the influenza infection on day 1 post infection in *Tmprss2^−/−^* and wild type mice with infected epithelial cells in the bronchiole (Figure 3A,B,E,F). However, at day 3 p.i. virus was spreading into the alveolar regions of wild type mice whereas it was only found in bronchioles in *Tmprss2^−/−^* mice (Figure 3C,D and G,H, respectively). Furthermore, infected wild type mice showed a strong increase in lung infiltrates and also in the number of infected cells whereas in *Tmprss2* knock-out mice a much lower number of infiltrating cells and infected cells were observed (Figure 3 K,L and O,P, respectively). Thus, the absence of TMPRSS2 largely protects animals from virus spread and virus induced pathogenesis.

**Figure 4:**
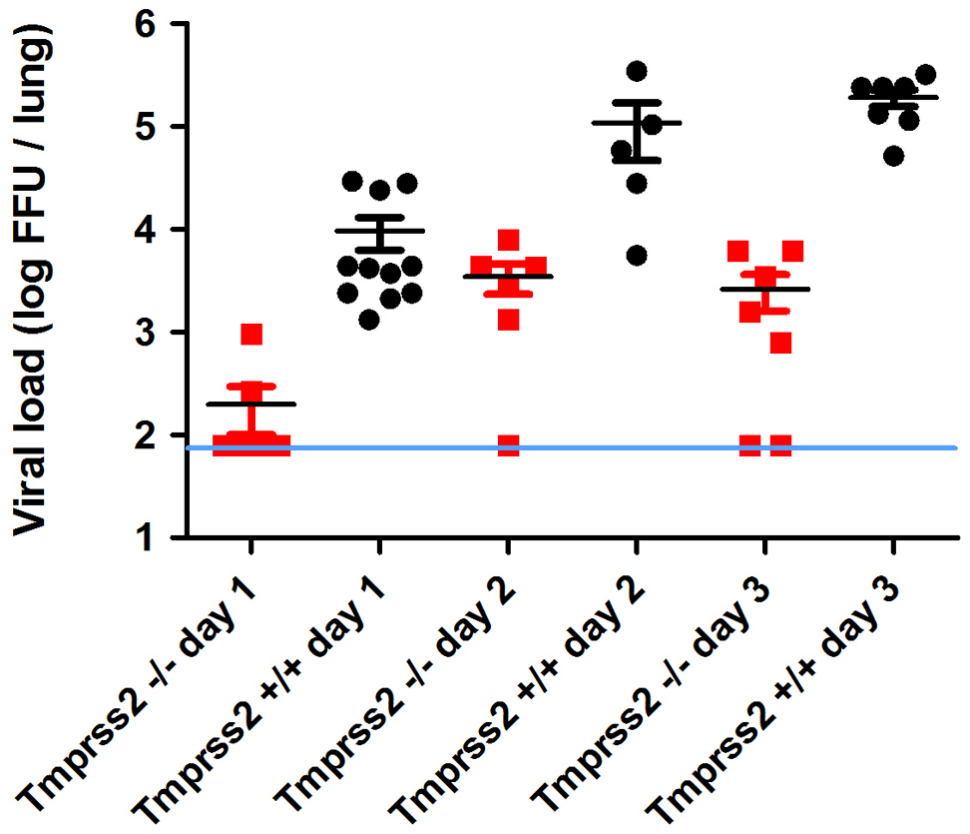
Viral load in the lungs of *Tmprss2^−/−^* mice after infection with H1N1 (PR8M) influenza A virus. Eight to eleven weeks old female mice were infected with 2×10^5^ FFU of the PR8M virus. Infectious virus particles were determined in lung homogenates. Viral load was higher in infected wild type mice compared to infected homozygous mutant mice at days 1, 2 and 3 p.i. Individual values, mean and SEM are presented. Detection limit of the assay is at 80 infectious particles per lung indicated by the blue line. Day 1 p.i. n = 9 for *Tmprss2^−/−^*, n = 11 for *Tmprss2^+/+^*, day 2 p.i. n = 6 for *Tmprss2^−/−^*, n = 5 for *Tmprss2^+/+^*, day 3 p.i. n = 7 for *Tmprss2^−/−^*, n = 7 for *Tmprss2^+/+^*.

Next, we assessed if protection from pathogenesis was due to reduced viral spread. After infection with PR8M, we could detect infectious viral particles in the lung in both homozygous *Tmprss2^−/−^* and wild type mice. However, the number of infectious particles was close to background at day 1 p.i. in *Tmprss2^−/−^* mice and was markedly reduced at days 2 and 3 p.i. compared to wild type mice (Figure 4). Influenza-specific antibodies were readily detectable in sera of *Tmprss2* knock-out mice (Figure S2) after infection with PR8M and PR8F virus demonstrating that the inoculated virus was able to infect lung cells and elicit a humoral immune response. In conclusion, these results show that TMPRSS2 is critical for efficient spread and pathogenesis of epidemic and pandemic H1N1 influenza viruses *in vivo*.

### Tmprss2 is required for HA cleavage in mouse lungs

**Figure 5:**
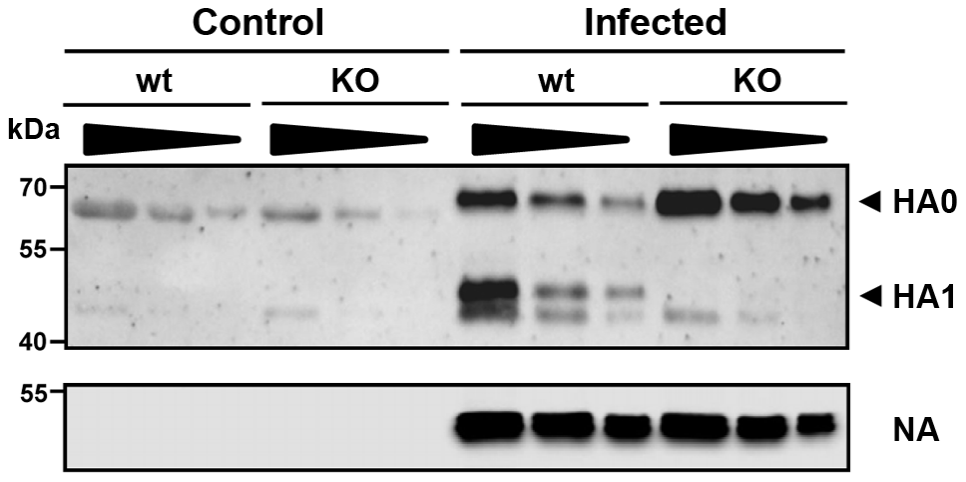
The hemagglutinin of H1N1 PR8M influenza virus is not processed in *Tmprss2* knock-out mice. BAL from infected wild type and *Tmprss2^−/−^* male mice was harvested at day 1 after infection with 2×10^5^ FFU PR8M and viral particles were concentrated by centrifugation through a sucrose cushion. As control, BAL from non-infected wild type and *Tmprss2^−/−^* was analyzed. Each sample was loaded undiluted (first lane), and in two dilutions (second lane 1∶1.33, third lane 1∶2). The virus-containing pellets were then analyzed for HA cleavage by Western blots. As loading control, the stripped membranes were incubated with anti-influenza A virus antibody confirming that equal amounts of proteins were loaded for respective undiluted and diluted samples.

Next, we sought to obtain direct evidence for the lack of proteolytic cleavage of HA in *Tmprss2* deficient mice. For this, broncho-alveolar lavages (BAL) of infected mice were collected and the proteolytic processing of the HA precursor protein HA0 was analyzed. After infection with a high dose of PR8M virus, processed HA1 as well as non-processed HA0 protein were detected in infected wild type mice whereas only HA0 protein was found in homozygous *Tmprss2* mutant mice (Figure 5). These results demonstrate that TMPRSS2 is essential for efficient HA cleavage activation in mice.

### *Tmprss2*-deficient mice were also protected from pathogenesis after infection with H3N2 virus but to a lesser extent

**Figure 6:**
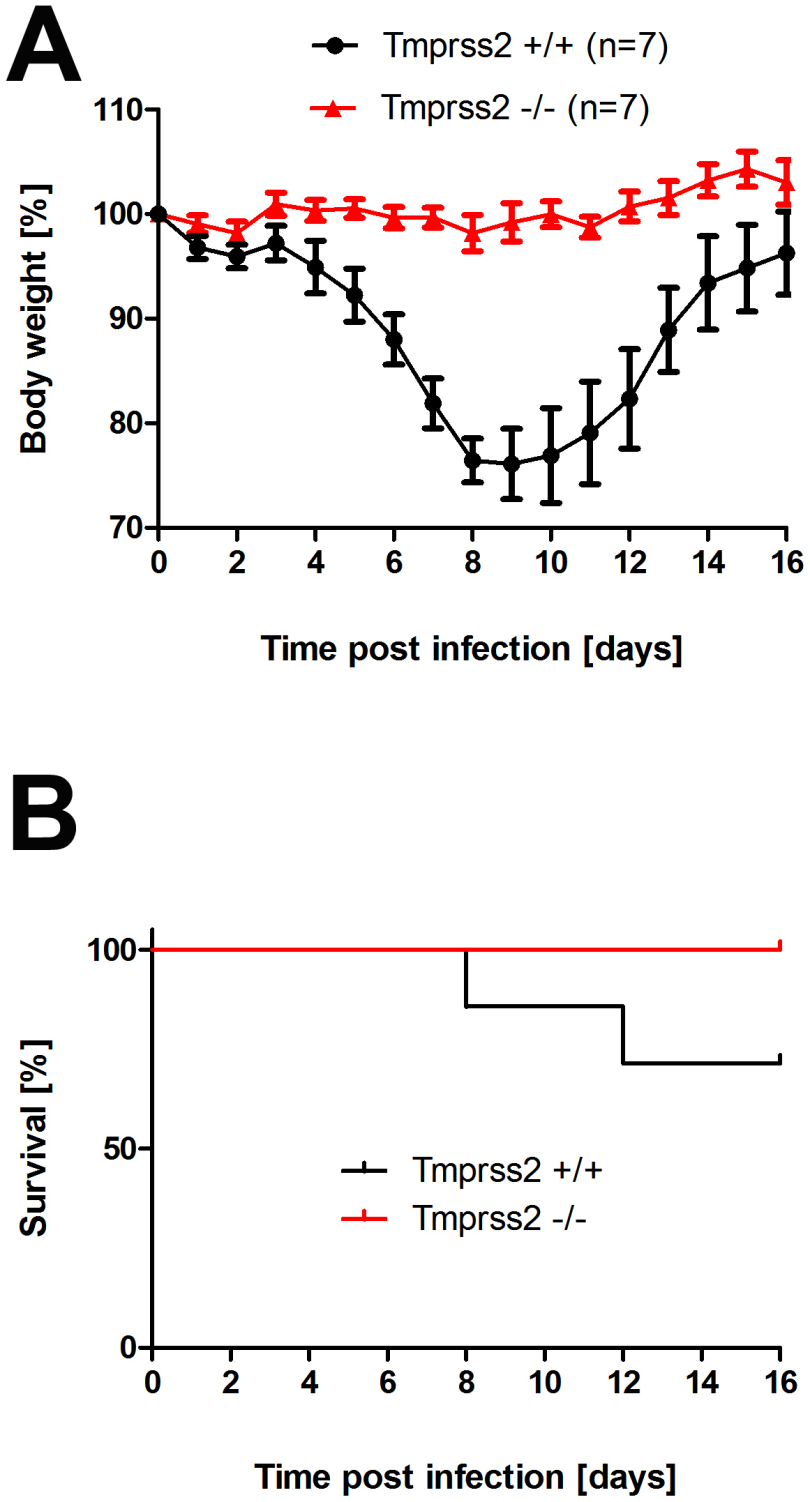
*Tmprss2* knock-out mice show reduced body weight loss and mortality after infection with low dose H3N2 influenza A virus infections. Eight to eleven weeks old female mice were infected with 10^1^ FFU mouse-adapted H3N2 influenza virus by intra-nasal application and bodyweight (A) and survival (B) was monitored until day 14 p.i. In addition to mice that were found dead, mice with a weight loss of more than 30% of the starting body weight were euthanized and recorded as dead. Homozygous *Tmprss2* knock-out mice lost significantly less weight than wild type (*e.g.* p = 0,0006 at day 7, and p = 0,0006 at day 8, using MWU test) mice and showed reduced mortality compared to wild type mice, although this difference was not significant (using the log rank test).

**Figure 7:**
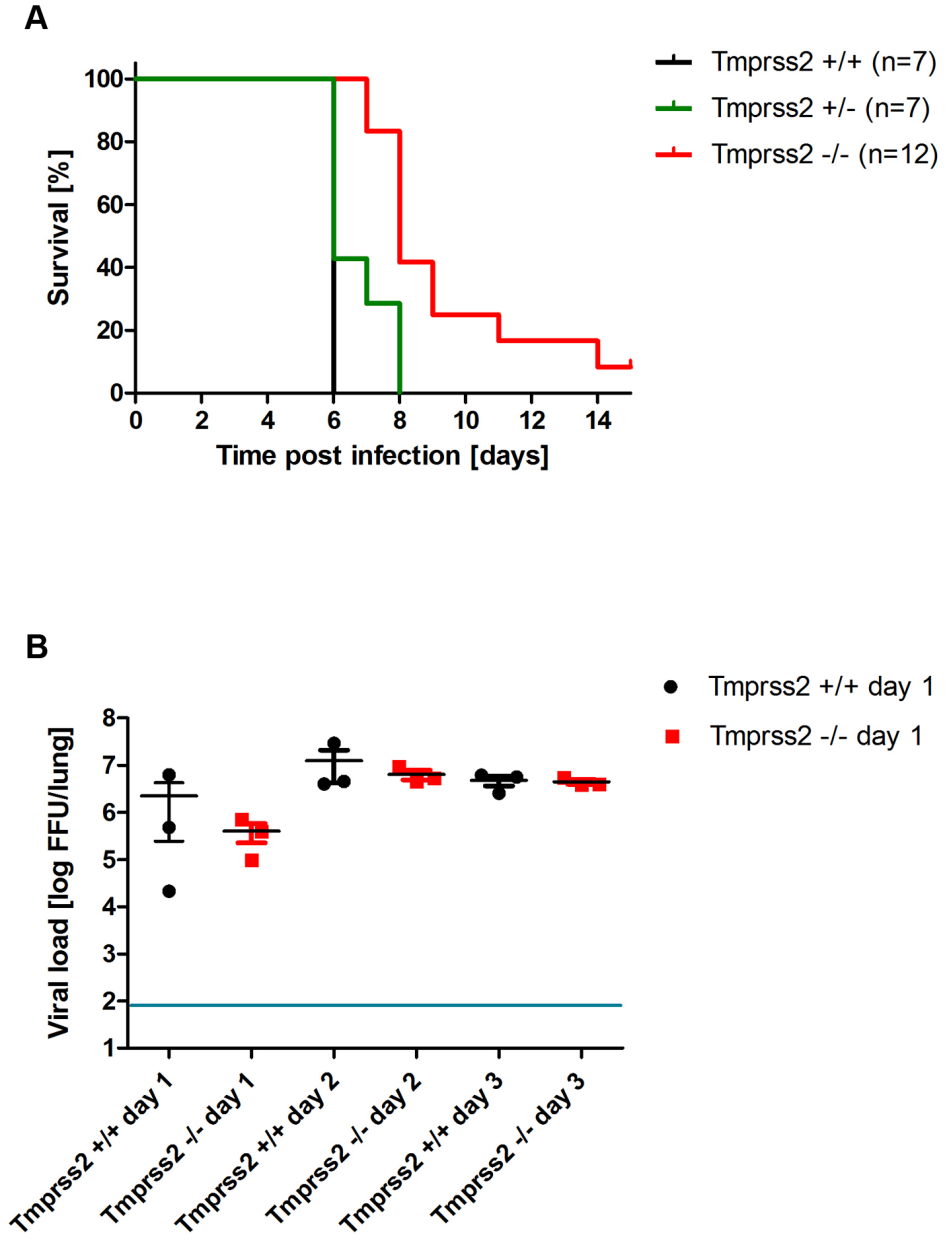
*Tmprss2* knock-out mice show reduced mortality after infection with high dose H3N2 influenza A virus infections. Eight to eleven weeks old female mice were infected with 2×10^3^ FFU mouse-adapted H3N2 influenza virus by intra-nasal application and survival (A) was monitored until day 14 p.i. In addition to mice that were found dead, mice with a weight loss of more than 30% of the starting body weight were euthanized and recorded as dead. Infectious viral particles were determined in lung homogenates (B). Individual values, mean and SEM are presented. Detection limit of the assay is at 80 infectious particles per lung indicated by the blue line. Homozygous *Tmprss2* knock-out mice showed significantly reduced mortality compared to wild type and heterozygote mice (p<0.0001 and p = 0.0032, respectively, using the log rank test). Viral load was not significantly different in infected wild type mice compared to infected homozygous mutant mice at days 1 to 3 p.i.

At present, influenza viruses of the HA subtypes H1 and H3 are circulating in humans. Therefore, we investigated if a H3 virus was also dependent on expression of a functional *Tmprss2* gene. After infection with a low dose (10^1^ Focus Forming Units (FFU)) of a mouse-adapted H3N2 virus (A/HK/01/68 [16]), body weight loss is less severe and survival is increased in *Tmprss2* knock-out compared to wild type mice (Figure 6A, B). After infection with a higher dose (2×10^3^ FFU) of H3N2 virus, mortality was significantly lower in *Tmprss2^−/−^* mice compared to wild type mice (Figure 7A). However, no significant differences were observed in the amount of infectious particles at day 1 to 3 p.i. (Figure 7B). Thus, activity of TMPRSS2 is required for the processing of both H1N1 and H3N2 but H3N2 viruses may be cleaved by other proteases in addition to TMPRSS2.

### Tmprss2 is not required for spread and pathogenesis of a virus with a multi-basic cleavage site

**Figure 8:**
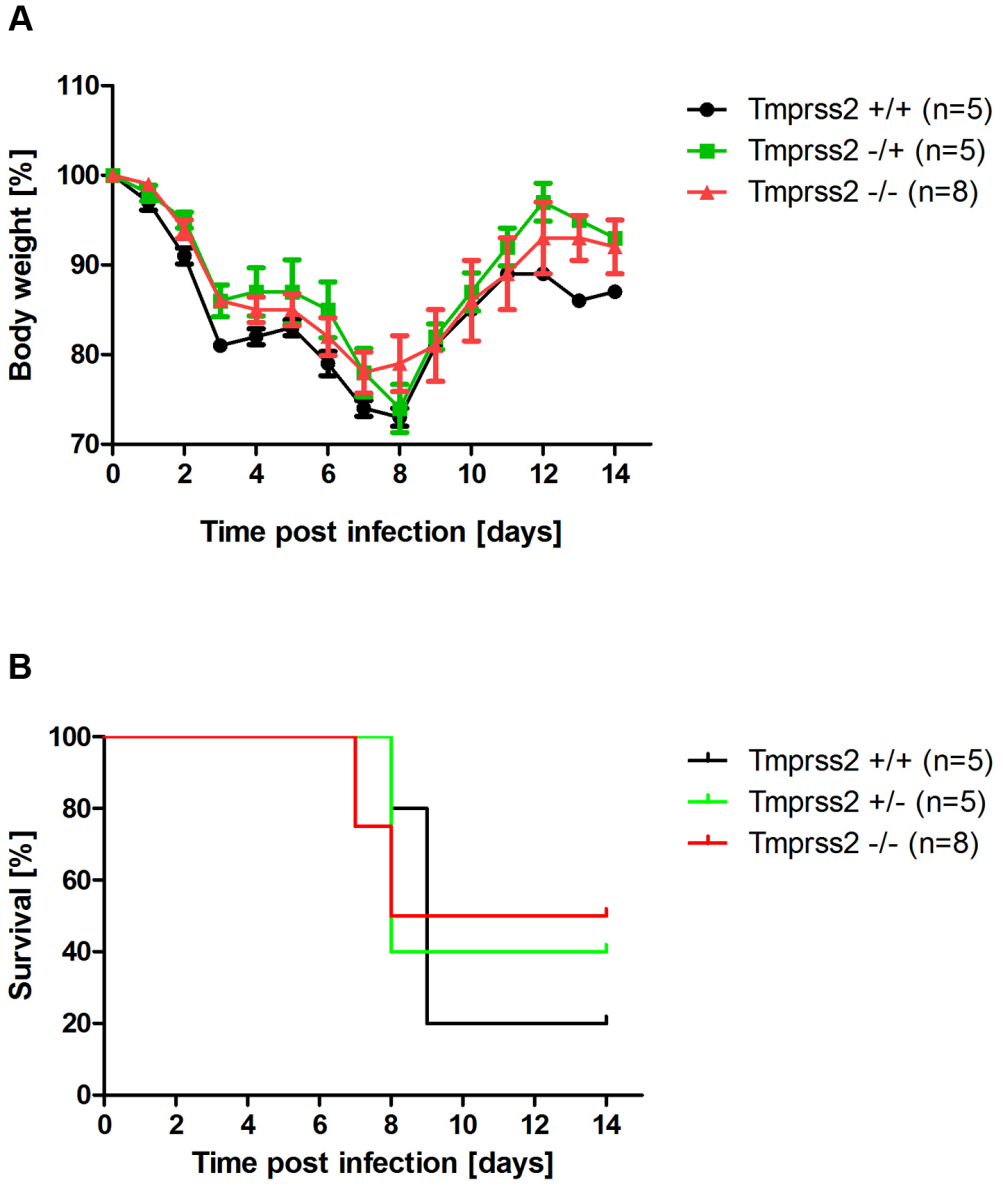
Murine Tmprss2 is not required for spread of H7N7 influenza A virus. Eight to eleven weeks old female mice were infected with 2×10^4^ FFU mouse-adapted SC35M (H7N7) influenza virus by intra-nasal application and bodyweight (A) and survival (B) was monitored until day 14 p.i. In addition to mice that were found dead, mice with a weight loss of more than 30% of the starting bodyweight were euthanized and recorded as dead. No significant differences were observed in survival between heterozygous and homozygous mutant mice after H7N7 infections (using the log rank test).

In cell culture, TMPRSS2 is dispensable for cleavage activation of viruses with a multi-basic cleavage site [10]. Thus, if the resistance of *Tmprss2* knock-out mice to H1N1 infection was indeed due to lack of HA processing, a virus with a multi-basic cleavage site should spread efficiently and cause disease. To investigate this, we infected mice with SC35M (mouse-adapted A/Seal/Massachusetts/1/80, H7N7) influenza virus which contains a multi-basic HA cleavage site. Mortality and body weight loss in infected *Tmprss2^−/−^* mice were not significantly different compared to wild type and *Tmprss2^+/−^* infected mice (Figure 8), suggesting that the presence of a multi-basic cleavage site renders viral spread and pathogenesis independent of TMPRSS2 expression.

### Cleavage-activation of diverse influenza viruses by murine Tmprss2 in vitro

Finally, we investigated if murine Tmprss2 was able to activate HA proteins of H1N1 and H3N2 viruses. The co-expression of protease and the respective HA proteins of PR8M, HA4, and H3N2 viruses in transfected cells facilitated HA cleavage of all viruses (Figure S3A). Additionally, expression of TMPRSS2 in this cell culture system allowed the spread of PR8M and HA4 viruses in a trypsin-independent fashion (Figure S3B). In contrast, spread of mouse-adapted SC35M virus, containing a multi-basic cleavage site did not depend on TMPRSS2 expression [10]. Thus, murine Tmprss2, like its human homologue [11], [17] can activate HA.

## Discussion

Cleavage activation of influenza virus HA by host cell proteases is essential for viral infectivity [2], [3]. However, the nature of the proteases required for the cleavage activation of viruses with a mono-basic HA cleavage site in the infected host organism remains unclear. At least eight candidate enzymes from different protease families have been suggested based on cell culture studies [18], leading to the concept that redundant proteolytic enzymes activate influenza viruses in the host. Here, we show for the first time that the deletion of a single protease, TMPRSS2, in mice largely abrogates viral spread and protects animals from severe pathology and death after H1N1 and, to a lower extent, H3N2 influenza virus infection.

After infection of *Tmprss2^−/−^* mice with H1N1 virus, no processing of the HA precursor protein HA0 was observed in BAL. However, an initial increase in viral titers was measured from day 1 to day 2 p.i. whereas at later times p.i. viral titers rapidly decreased. This initial increase in viral titers is expected because the virus used for infections had been produced in embryonated chicken eggs. It therefore carries an activated HA allowing it to enter cells and replicate [19], [20]. In addition, it is conceivable that other proteases besides TMPRSS2 may facilitate low levels of H1 cleavage and allow limited viral spread which is rapidly cleared once the antiviral immune responses have been activated. However, the cleavage activation by such alternative enzymes must be very inefficient since viral titers in H1N1 infected *Tmprss2^−/−^* mice were markedly reduced compared to wild type animals and no weight loss was observed.

**Figure 9:**
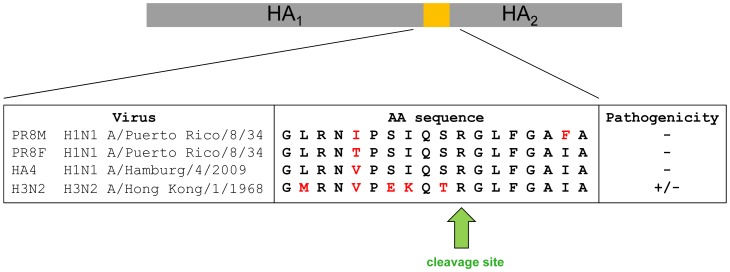
Alignment of amino acid sequences of the protease loop region from H1N1 and H3N2 influenza A viruses. Pathology is strongly reduced in *Tmprss2^−/−^* mice after infection with H1N1 virus and diminished after infection with H3N2 virus.

Surprisingly, H3N2 virus which also carries a mono-basic cleavage site in the HA, was able to replicate in *Tmprss2^−/−^* mice. However, body weight loss and mortality were significantly reduced in knock-out mice compared to wild type mice in a dose-dependent manner. On the other hand, viral load was not significantly lower in mutant mice after infection with 2×10^3^ FFU. The amino acid sequence in the HA loop which is recognized by proteases differs between HA subtypes H1 and H3 (Figure 9). A recent study demonstrated that different proteases, including TMPRSS2, TMPRSS11d (HAT) and even trypsin, cleave HA from different subtypes and variants with varying efficiency [21]. In addition, ST14 (matriptase) has the capability to cleave HA of particular H1 subtype strains but only minimal cleavage was observed for H2 and H3 [22]. Furthermore, KLK5 and 12 (kallikrein related-peptidase 5 and 12) have been described as host proteases that are capable of cleavage activation of viral H1, H2 and H3 HA *in vitro* [23]. Thus, H3N2 appears to be processed to some extent by TMPRSS2, resulting in the reduced pathology in *Tmprss2* knock-out mice but also other proteases are able to cleave H3 hemagglutinin *in vivo*. Finally, it is noteworthy that *Tmprss2^−/−^* mutant mice were not protected from infection with an H7N7 virus which contains a multi-basic HA cleavage site and can be activated by ubiquitously expressed proteases [4].

Our findings may have potential for the development of future influenza virus therapeutics. Broad spectrum protease inhibitors have been shown to inhibit influenza virus in cell culture and *in vivo* [24], [25], [26], [27], [28] but unwanted side effects are a major concern. The results reported here suggest that targeting a single protease, TMPRSS2, may be sufficient to achieve a notable therapeutic benefit against H1N1 influenza viruses and possibly other subtypes. Blocking of TMPRSS2 may not be associated with severe unwanted side effects, since *Tmprss2^−/−^* mice are healthy and do not show any phenotypic alterations in the absence of an infection [14]. Furthermore, TMPRSS2 inhibitors might exert activity against diverse respiratory infections. For example, human metapneumovirus [29], the emerging MERS-coronavirus [30], [31] and SARS-coronavirus [32], [33], [34] can also be activated by TMPRSS2 in cell culture and might use this protease to support their spread in the infected host. It should, however, be noted that our findings in the mouse model system require validation in humans.

## Materials and Methods

### Ethics statement

All experiments in mice were approved by an external committee according to the national guidelines of the animal welfare law in Germany (‘Tierschutzgesetz in der Fassung der Bekanntmachung vom 18. Mai 2006 (BGBl. I S. 1206, 1313), das zuletzt durch Artikel 20 des Gesetzes vom 9. Dezember 2010 (BGBl. I S. 1934) geändert worden ist.’). The protocol used in these experiments has been reviewed by an ethics committee and approved by the ‘Niedersächsisches Landesamt für Verbraucherschutz und Lebensmittelsicherheit, Oldenburg, Germany’ (Permit Number: 33.9.42502-04-051/09).

### Virus, mice and plasmids

Original stocks of viruses were obtained from Stefan Ludwig, University of Münster (PR8M, A/PuertoRico/8/34 H1N1, Münster variant), from Peter Stäheli, University of Freiburg (PR8F, A/PuertoRico/8/34 H1N1, Freiburg variant and SC35M [35]), from Otto Haller, University of Freiburg (H3N2, [16]) and from Thorsten Wolff, Robert-Koch-Institute, Berlin (HA4). The different virulence of PR8M and PR8F virus isolates has been described before [15]. SC35M (H7N7) was originally derived from the seal, adapted to the mouse by serial passages in the mouse lung [35], and the laboratory strain which was used here was generated by reverse genetics using the plasmid rescue system [36]. Virus stocks of PR8 were prepared by infection of 10-day-old embryonated chicken eggs and for HA4 on MDCK cells as described [37]. Mutant *Tmprss2^−/−^* mice were on a mixed C57BL/6J – 129 background [14]. Animals were maintained under specific pathogen free conditions at the animal facility of the HZI in Braunschweig. Heterozygous mutant mice were interbred and wild type, heterozygous and homozygous mutant mice were genotyped by PCR analysis and then used for infections. Genotyping of *Tmprss2* alleles was carried out using a three primer strategy (P1 5′TGTGCCCTTGGACAGATGACTC3′, P2 5′GGACTACAGATATGAGGTGTTC3′, P3 5′AGGCCAGAGGCCACTTGTGTAG3′) that allows to distinguish between wild type (yielding a 540 bp product) and knock-out (yielding a 400 bp product) alleles, respectively. Expression plasmids encoding human and mouse proteases TMPRSS2 were published earlier [10], [13]. Previously described expression plasmids for PR8M [38] were used as templates for amplification of the respective coding regions using oligonucleotides PR8-HA-5Acc: GGGGGTACCACCATGAAGGCAAACCTACTGGTCCTG, PR8-HA-3Nhe: GGGCGCTAGCTCAGATGCATATTCTGCACTG. The resulting PCR products were inserted into plasmid pCAGGS using the *Acc65I* and *Nhe*I sites. For cloning of HA4 HA protein, expression plasmid kindly provided by Prof. Klenk [39] was used as templates for amplification of the coding region using oligonucleotides swi09-HA-5Eco: GGGAATTCACCATGAAGGCAATACTAGTAGTTCTGC and swi09-HA-3Xho: GGGCTCGAGTTAAATACATATTCTACACTGTAGAG. The resulting PCR products were inserted into plasmid pCAGGS using *EcoRI* and *XhoI*.

### Infection of mice and measurement of body weight loss and survival

For infection experiments, female mice at the age of 8–11 weeks were anesthetized by intra-peritoneal injection of Ketamin-Xylazine solution in sterile NaCl (50 mg/ml Ketamine, Invesa Arzneimittel GmbH, Freiburg; 2% Xylazine, Bayer Health-Care, Leverkusen) with a dose adjusted to the individual body weight. Infection was performed by intranasal application of virus solution in 20 µl of sterile phosphate-buffered saline. Subsequently survival and body weight loss were monitored until day 14 p.i. In addition to mice that were found dead, mice with a weight loss of more than 30% of the starting body weight were euthanized and recorded as dead.

### Determining infectious viral particles

Viral load in infected lungs was determined on MDCK II (Madin-Darby Canine Kidney II) cells using the FFU assay as described [15]. Detection limit of the assay is at 80 infectious particles/lung. Thus, for samples where no virus was detected, the data points were set to 80 FFU/lung.

### Analysis of processing of HA from infected lungs

For analysis of HA in viral particles, Broncho-alveolar lavages (BALs) of wild type and *Tmprss2* knock-out mice infected with 2×10^5^ FFU PR8M were harvested at day 1 p.i., centrifuged at full speed and supernatant were loaded on a 20% sucrose cushion for concentration of viral particles for 2–3 h at full speed and 4°C. The pelleted viral particles were lysed in 2×SDS loading buffer. For immuno-blotting, the lysates were separated by SDS gel electrophoresis, blotted on a nitrocellulose membrane and HA was detected by staining with a rabbit anti-PR8HA antibody (Sino biological) at a dilution of 1∶500, followed by incubation with a horseradish peroxidase (HRP)-coupled anti-rabbit antibody (Dianova) at a dilution of 1∶10.000. As loading control, membranes were stripped (Stripping Buffer: 1M Tris-HCl (pH 6,8), 10% SDS, 100 mM ß-Mercaptoethanol) for 30 min at 50°C, washed 1 h with dH_2_O and stained for NA by using an anti-influenza A virus goat serum (Millipore) at a dilution of 1∶500 followed by incubation with a horseradish peroxidase (HRP)-coupled anti-goat antibody (Dianova) at a dilution of 1∶10.000. Bands were visualized by using a commercially available kit ECL Prime Western Blotting Detection Reagents (Amersham).

### Pulsoxymetric determination of oxygen saturation

Eight to twelve weeks old mice were infected intranasally with 2×10^5^ FFU PR8M and the amount of oxygen saturation was determined by the MouseOx® system (STARR Life Science Corp.) over a period of 14 days. For oxygen measurements mice were anesthetized using an isoflurane inhalator.

### Histological and immunohistochemical analyses

Lungs were prepared and immersion-fixed for 24 hours in 4% buffered formaldehyde solution (pH 7.4), dehydrated in a series of graded ethanol and embedded in paraffin. Sections (0.5 µm) were cut from five evenly distributed levels of the paraffin blocks and stained with haematoxylin and eosin. For immunohistochemical studies, sections were stained with a polyclonal primary antibody (against influenza A H1N1 virions; Virostat) overnight at 4°C and subsequently tissue sections were incubated for 30 min with secondary antibody (rabbit anti-goat-biotin; KPL; Gaithersburg, Madison, USA) and counterstained with haematoxylin.

### Influenza virus cleavage activation by TMPRSS2 in cell culture

293T cells were transiently transfected with expression plasmids encoding human or mouse TMPRSS2 or control transfected with empty plasmid. At 24 h post transfection, cells were infected with either of the influenza viruses PR8M, HA4 or SC35M at a multiplicity of infection (MOI) of 1. After 1 h incubation at 37°C, virus was removed and fresh MEM medium (supplemented with 0.2% BSA and 1 µg/ml TPCK-trypsin or PBS) was added to the cells. At 48 h p.i., supernatants were harvested, cleared from debris by centrifugation for 5 min at 3.500 rpm and stored at −80°C until quantification of infectious virus particles by focus formation assay (FFU) as described previously [15].

## Supporting Information

Figure S1 **Tmprss2 is essential for H1N1 influenza virus pathogenesis.** Eight to eleven weeks old female mice were infected with 2×10^5^ FFU mouse-adapted PR8M (H1N1). Survival was monitored until day 14 p.i. In addition to mice that were found dead, mice with a weight loss of more than 30% of the starting bodyweight were euthanized and recorded as dead. All *Tmprss2* knock-out mice survived the infections whereas about 50% of wild type or *Tmprss2* heterozygous mice died.
(TIF)

Figure S2 **Surviving** ***Tmprss2^−/−^*** **mice mount antibodies against viral proteins after infection with H1N1 influenza A virus.** After infection, blood from surviving mice was collected by heart puncture. Sera were diluted 1∶1000 and an ELISA was performed using plates that were coated with 1.6×10^5^ FFU PR8M virus. For detection of virus specific IgG, peroxidase-labeled anti-mouse IgG (KPL, Gaithersburg, Madison, USA) was used as a secondary antibody and visualization of the reaction was carried out using a peroxidase specific substrate. Absorbance at 490 nm is shown. As control, sera from non-infected mice were analyzed. Sera from surviving wild type and homozygous *Tmprss2* mutant mice infected with PR8M (A) or PR8F (B) were analyzed 14 days after infection for influenza-specific IgG antibodies. Individual values, mean and SEM are presented.
(TIF)

Figure S3 **Murine Tmprss2 activates diverse H1N1 influenza viruses by cleavage of the hemagglutinin.** The indicated HA proteins (PR8M, HA4 and H3N2) and proteases (human or mouse TMPRSS2) were transiently co-expressed in 293T cells, the cells were treated with PBS or trypsin, and HA cleavage was detected by Western blotting (A). Protease transfected 293T cells were infected with the indicated viruses at a multiplicity of infection of 1 in the presence or absence of trypsin. At 48 h p.i., viral spread was quantified by focus formation assay (B). Virus release into the medium is presented as means ± SD and was confirmed in at least two independent experiments.
(TIF)
