# Correction: Tmprss2 Is Essential for Influenza H1N1 Virus Pathogenesis in Mice

**DOI:** 10.1371/journal.ppat.1004435

**Published:** 2014-09-10

**Authors:** The PLOS Pathogens Staff

The name of the second to last author in the authorship listing is spelled incorrectly. The correct spelling of the name is: Stefan Pöhlmann. The citation from the original article remains the same.
