# Supplementary figures and images for: Tmprss2 Is Essential for Influenza H1N1 Virus Pathogenesis in Mice
Source: PLoS Pathog. 2013 Dec 5;9(12):e1003774. doi: 10.1371/journal.ppat.1003774 (PMC3857797; doi:10.1371/journal.ppat.1003774)

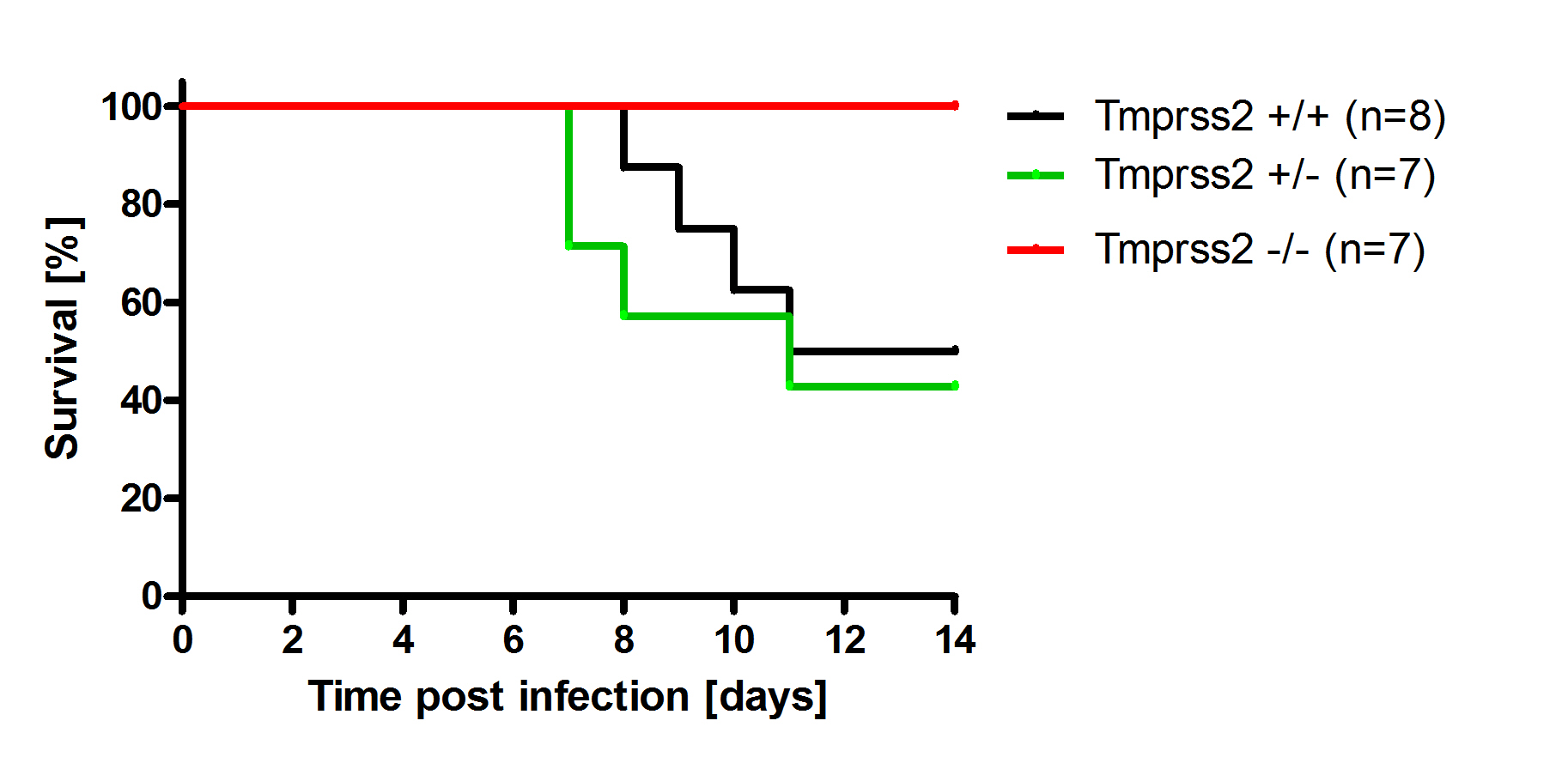

Supplement: Figure S1 — Tmprss2 is essential for H1N1 influenza virus pathogenesis. Eight to eleven weeks old female mice were infected with 2×10^5^ FFU mouse-adapted PR8M (H1N1). Survival was monitored until day 14 p.i. In addition to mice that were found dead, mice with a weight loss of more than 30% of the starting bodyweight were euthanized and recorded as dead. All Tmprss2 knock-out mice survived the infections whereas about 50% of wild type or Tmprss2 heterozygous mice died. (TIF) [file ppat.1003774.s001.tif]

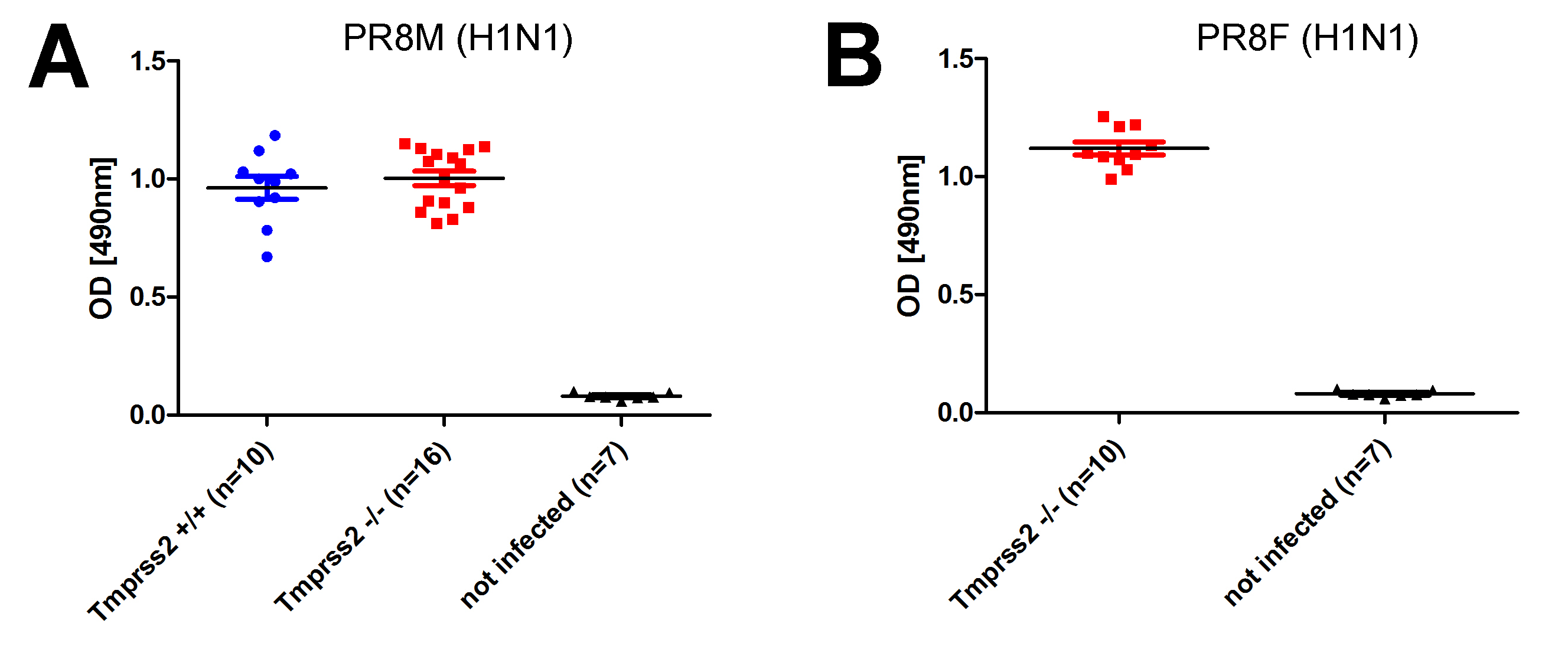

Supplement: Figure S2 — Surviving Tmprss2−/− mice mount antibodies against viral proteins after infection with H1N1 influenza A virus. After infection, blood from surviving mice was collected by heart puncture. Sera were diluted 1∶1000 and an ELISA was performed using plates that were coated with 1.6×10^5^ FFU PR8M virus. For detection of virus specific IgG, peroxidase-labeled anti-mouse IgG (KPL, Gaithersburg, Madison, USA) was used as a secondary antibody and visualization of the reaction was carried out using a peroxidase specific substrate. Absorbance at 490 nm is shown. As control, sera from non-infected mice were analyzed. Sera from surviving wild type and homozygous Tmprss2 mutant mice infected with PR8M (A) or PR8F (B) were analyzed 14 days after infection for influenza-specific IgG antibodies. Individual values, mean and SEM are presented. (TIF) [file ppat.1003774.s002.tif]

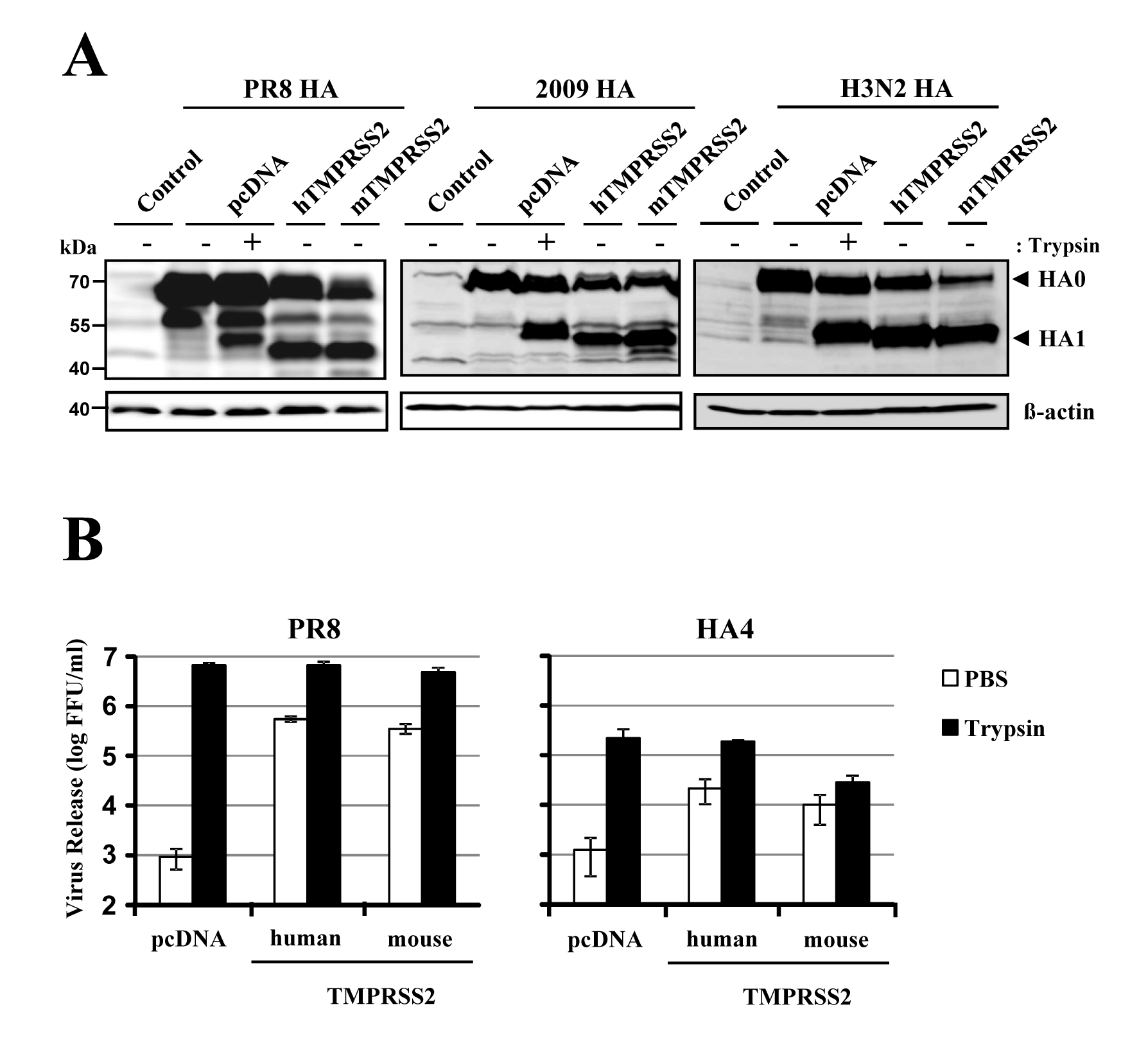

Supplement: Figure S3 — Murine Tmprss2 activates diverse H1N1 influenza viruses by cleavage of the hemagglutinin. The indicated HA proteins (PR8M, HA4 and H3N2) and proteases (human or mouse TMPRSS2) were transiently co-expressed in 293T cells, the cells were treated with PBS or trypsin, and HA cleavage was detected by Western blotting (A). Protease transfected 293T cells were infected with the indicated viruses at a multiplicity of infection of 1 in the presence or absence of trypsin. At 48 h p.i., viral spread was quantified by focus formation assay (B). Virus release into the medium is presented as means ± SD and was confirmed in at least two independent experiments. (TIF) [file ppat.1003774.s003.tif]
